# The British Dental Association and the National Dental Association of the United States Contrasted

**Published:** 1902-11-15

**Authors:** William H. Trueman

**Affiliations:** Philadelphia


### THE BRITISH DENTAL ASSOCIATION AND THE NATIONAL DENTAL ASSOCIATION OF THE UNITED STATES CONTRASTED.

BY WILLIAM H. TRUEMAN, D.D.S., PHILADELPHIA.

The *Dental Record* (London) for July, 1902, contains a report, covering some nineteen pages, of the last meeting of the British Dental Association, at Shrewsbury, May 22 to 24, 1902. While it is a mere *resume*, it is interesting and in regard to some matters invites careful thought. In the first place, we are informed that the Association has 1,205 members, that during the past year eight only have resigned and thirteen names have been dropped for non-payment of dues. This is a remarkable showing. It demonstrates the keen interest the dental profession of Great Britain takes in its National Association, and their loyalty. The loss of only thirteen from non-payment of dues for two or more years out of a membership of 1,205 is a record of which but few dental societies can boast.

Now, why is it that in England so large a proportion of qualified dentists are members of the National Association, while in the United States the National Association is so poorly supported, and numbers so few? If our national society was in practice, as it is in theory, a representative body, the disparity would be seeming only. It is not so; its complex arrangement of permanent members and delegates makes the provision limiting representation of affiliating organizations practically null; for the so-called election of delegates that is annually gone through with by the bodies recognized by the National Association soon gives each one an opportunity, so that, notwithstanding the rule, every member of a local society may be a member of the National if he so choose. Only about one in fifty legal practitioners in the United States avail themselves of this wide-open door, while in England the proportion is about one in five or six. Geographical conditions may explain this in part, but is it a full answer?

There is another matter. In the report referred to we are informed that the British Dental Association has an invested fund of £2,000 (about $9,600); to this £300 are to be added; in addition to which it has £200 in its banker's hands. In addition and apart from this fund it maintains a benevolent fund,* with a large and constantly increasing sum at interest, notwithstanding that its trustees from its yearly income are contributing to the support of disabled members of the profession, their

* Donations and collections for the benevolent fund, £212; amount of investment, £2,192; paid to beneficiaries, £648 ($3,110).—*Report* 1902.

widows and children. Quietly, that benevolent fund has done and is still doing a world of good. It has made comfortable the last years of worthy but unfortunate practitioners, regardless of whether they are members of the Association or not; it has stepped in at the right time, and in the right way, to assist the widows of those who have fallen by the wayside, and has cared for and educated their children.

In addition to all this, the British Dental Association has for years published a monthly dental journal all its own, absolutely free from trade entanglements of any kind or character. In its beginning this journal, as a dental journal, did not amount to much; it was a serious expense; while each year the deficiency became less, it continued for many years a heavy drain upon the finances of the Association to make good the difference between the income and the expenses of the journal. The time came, however, when they balanced; when the *Journal* began to be a source of profit; then it expanded, took on a new character; and to-day it is the peer of any dental journal in the world. In the meantime it had been a bond of union between the local societies and the national body, contributing to the success of each. In the beginning it was little more than a medium for announcing dental society business, but as such was useful to those who desired to keep in close touch with that phase of professional progress. It still is the recognized official organ of the dental profession in Great Britain, and still retains its usefulness of keeping in close touch the local and national associations by being the medium through which their various announcements are made to the profession, and through which much of their proceedings are published; but in addition to this, in its present form it monthly publishes a large amount of valuable original matter and a judicious selection of translations from foreign dental journals which otherwise would be unknown to English readers. Its large circulation has secured an extensive and varied advertising patronage, adding not only to the profits of its publication, but also to its value as a professional journal.

Its monthly visits are a constant reminder of the existence and usefulness of the British Dental Association, and keeps its readers, whether members or not, well informed of all its doings, financial and otherwise; it takes them into its confidence and invites their interest; as a result, the Association has some 1,200 members, about half of whom annually attend its meetings.

In contrast to this, our National Association has no official organ, but trades its proceedings to whoever makes the best bid. The successful journal selects such of its proceedings as suits its purpose, and dribbles them out month by month, as suits its convenience. Its proceedings in full are for its members only, and are as inaccessible to outsiders as are the secrets of a Masonic lodge; not a volume can be obtained for love or money. It has no interest in, nor does it in any wise assist the local societies, nor does it do anything more for the profession at large than do many State and local societies. None outside the circulation of the favored journal are reminded of its existence from one year's end to the other. As a natural result, out of the twenty or twenty-five thousand legal practitioners in the United States, a beggarly few hundred only are members, or take in it the slightest interest. It offers no inducement whatever to those unable to attend its meetings to add to its funds or influence by becoming members.—*International.*

